# Crossing of the intestinal barrier by HTLV-1 infected lymphocytes

**DOI:** 10.1186/1742-4690-12-S1-O33

**Published:** 2015-08-28

**Authors:** Florent Percher, Patricia Jeannin, Pierre-Guillaume Deliège, Antoine Gessain, Aurore Vidy-Roche, Philippe V Afonso, Pierre-Emmanuel Ceccaldi

**Affiliations:** 1 Institut Pasteur, Unité d'Epidémiologie et Physiopathologie des Virus Oncogènes, Paris, France; 2 CNRS, UMR 3569, Paris, France; 3 Université Paris Diderot, Cellule Pasteur, Paris, France

## 

In several areas of high endemicity, HTLV-1 can be transmitted from mother to child, through prolonged breast-feeding. This way of transmission is particularly linked to the development of Adult T Cell Leukemia (ATL). In this context, we studied the mechanisms of HTLV-1 transmission through the digestive tract. We previously demonstrated in an in vitro model of compartimentalized co-culture device of human enterocytes, lymphocytes and dendritic cells, that HTLV-1 was unable to infect enterocytes, or disrupt tight cell junctions, but could rather be transported by transcytosis to infect sub-epithelial dendritic cells (Martin-Latil et al., Blood, 2012). Since HTLV-1 infection has been shown to be more efficient through cell-associated virions, we also focused on the crossing of HTLV-1 infected lymphocytes through the intestinal barrier. We thus studied the migration of HTLV-1 infected lymphocytes across the intestinal epithelium, both ex vivo (on intestinal explant model) and in vitro (on a monolayer of human enterocytic cell line cultured on Transwell devices). We demonstrate that, in both systems, infected lymphocytes are able to cross the epithelial barrier, with increased capacities compared to uninfected lymphocytes. Our work suggests that HTLV-1 infected lymphocytes efficiently cross the intestinal barrier, which could constitute an additional way for mother to child HTLV-1 transmission during breast-feeding.

